# Change in Spatial Distribution Patterns and Regeneration of *Populus euphratica* under Different Surface Soil Salinity Conditions

**DOI:** 10.1038/s41598-019-42306-7

**Published:** 2019-06-24

**Authors:** Pei Zhang, Xiaoya Deng, Aihua Long, Hailiang Xu, Mao Ye, Junfeng Li

**Affiliations:** 1 State Key Laboratory of Simulation and Regulation of Water Cycle in River Basin, Department of Water Resources, China Institute of Water Resources and Hydropower Research, Beijing, 100038 China; 2 State Key Laboratory of Desert and Oasis Ecology, Xinjiang Institute of Ecology and Geography, Chinese Academy of Sciences (CAS), Urumqi, 830011 China; 3 School of Geography Science and Tourism, Xinjiang Normal University, Urumqi, 830054 China; 4 College of Water Conservancy and Architectural Engineering, Shihezi University, Shihezi, 832003 China

**Keywords:** Forest ecology, Conservation biology

## Abstract

Ecological conservation and restoration have increasingly captured attention worldwide due to the degradation of ecosystems. As one of the most ecologically fragile areas, the Tarim River Basin, of Xinjiang, China, encountered serious decline of desert riparian forests. The Chinese government has implemented the “Ecological Water Conveyance Project” (EWCP) since 2000, protecting and restoring the dominant natural species of the desert riparian forests, i.e., *Populus euphratica* Oliv. The regenerative effect after the water conveyance was noteworthy. For the purpose of clarifying the mechanism of *P*. *euphratica* forest regeneration to find a better prescription for the ecological restoration works in the Tarim River Basin, we investigated the relationship between the distribution of *P*. *euphratica* and soil salinity. Experimentally evaluated the effects of surface soil salinity on *P*. *euphratica* seed germination and the influence of river flooding on the salinity of surface soils. The results showed that (1) *P*. *euphratica* trees mainly spread along the river channel within 2 km; with increasing vertical distance to the channel, the number of trees declined significantly; (2) where the salinity of the surface soil is high, there are less living *P*. *euphratica* trees; (3) the germination of *P*. *euphratica* seeds decreases with increased soil conductivity; when the soil conductivity was higher than 7 ms/cm, the germination of *P*. *euphratica* seeds was severely curtailed. (4) Flooding regimes were a pre-condition of *P*. *euphratica* restoration; they had profound effects on improving the germination of the seeds via ameliorating water conditions and reducing salinity. Our results point out that the most efficient ecological prescription for restoring and protecting desert riparian forests is to induce flooding twice yearly during June to August with 10- to 15-day durations each time. Such a plan (especially in the Tarim River Basin) should prioritize the protection of seedlings.

## Introduction

Human disturbance, caused by population growth, development of the social economy and highly intensive use of resources, is becoming a global problem that directly or indirectly leads to the degradation of ecosystems and ecological environments^1–3^. The most marked effects are the decreases in primary and secondary productivity of ecosystems, the reduction or loss of biodiversity, and the invasion of exotic species^4–8^. The degradation of the ecological environment has seriously threatened the sustainable development of human beings. Since the 1960s, in order to mitigate and prevent the degradation of natural ecosystems, rehabilitation and reconstruction of damaged ecosystems have increasingly drawn attention from international communities. The purpose of the “Man and the Biosphere Program” (MAB) advocated by UNESCO is to use an ecological approach to study the relationship between human beings and the environment, especially the impact of human activities on ecosystems, and the management, utilization and recovery of resources under the influence of human activities^9–12^.

Influenced by the global trend, governments are aware of the seriousness of ecological degradation and the urgency of carrying out ecological restoration^13,14^. Some ecological restoration programs are implemented to mitigate ecological degradation, e.g. the “Reforestation of Fragmented Forests Project” in Kianjavato, Madagascar^15^, the “Megadiversity Atlantic Forest Biome Restoration Project” in Iracemápolis, Brazil^16^ and the “Comprehensive Management” in the Tarim River Basin^17^. In addition, flooding disturbance, taken from restoration ecology and the intermediate disturbance hypothesis, is implemented in many places to accomplish ecological restoration goals^18–22^. The flooding disturbance research focuses mainly on its changes in soil physical and chemical properties, community reconstruction and biodiversity variation, and ecological responses of plants to flooding. However, in most of these studies, are located in humid or sub-humid areas; whether this is suitable in arid areas has not been well studied.

The Tarim River Basin sustains the world’s largest contiguous natural *Populus euphratica* forest, accounting for 54% of the global *P*. *euphratica* growth area^23,24^. With great adaptive abilities (such as its tolerance to drought and salinity), *P*. *euphratica* is the major tree and keystone species that constitutes the desert riparian forest and forms a natural eco-barrier or so-called “green corridor.” It protects the development of oases, stabilizes river channels, maintains the ecological balance of river basins, and fertilizes forest soil^25–27^. Additionally, the desert riparian *P*. *euphratica* forest in the lower reaches of the Tarim River has other significant effects, separating the Kuluc Desert from the Taklamakan Desert and protect the artificial oases from invasion of wind-blown sand^28^. Due to high human use of the Tarim water resource, the river flow was drying-up in the lower reaches of the Tarim from the 1970s to 2000s, large areas of *P*. *euphratica* forest have been severely affected^29^; areas far away from the river channel have almost died out entirely. The remaining mature and over mature trees account for over 80%, and the young trees were no more than 15%, showing a declining structure. To rescue the area from the severe human settlement conditions, improve the fragile ecological environment, and protect the desert riparian *P*. *euphratica* forest, the Chinese government has spent 10.7 billion yuan on the “Ecological Water Conveyance Project” (EWCP) since 2000^17^. The EWCP transfers water from Boston Lake and the Tarim River to the lower reaches for ecological consumption. As of 31^th^ Oct. 2018, the total amount of water conveyances has reached 7.44 billion m^3^.

To preserve and regenerate the desert riparian *P*. *euphratica* forest in the lower Tarim area, the ecological and physiological characteristics of *P*. *euphratica* trees under drought stress were researched to explore the threshold groundwater depth for healthy *P*. *euphratica* growth^26,30–32^. Some others have researched the genetic expression^33–35^, salt, water and nutrient transport^29,36–39^ and photosynthesis^40,41^ on a micro-scale. Our previous research has revealed the minimum groundwater depths for different ages of *P*. *euphratica* trees in detail^24^. All these studies mainly focused on the effects of the water conditions on *P*. *euphratica* trees following the procedure of the river flow diversion and extremely severe water shortage in the lower reaches of the Tarim River. It should be noticed that the salinity of the soil in this area is high, and soil salinity may also play an essential role, taking influence in the process of *P*. *euphratica* growth.

**Figure 1:**
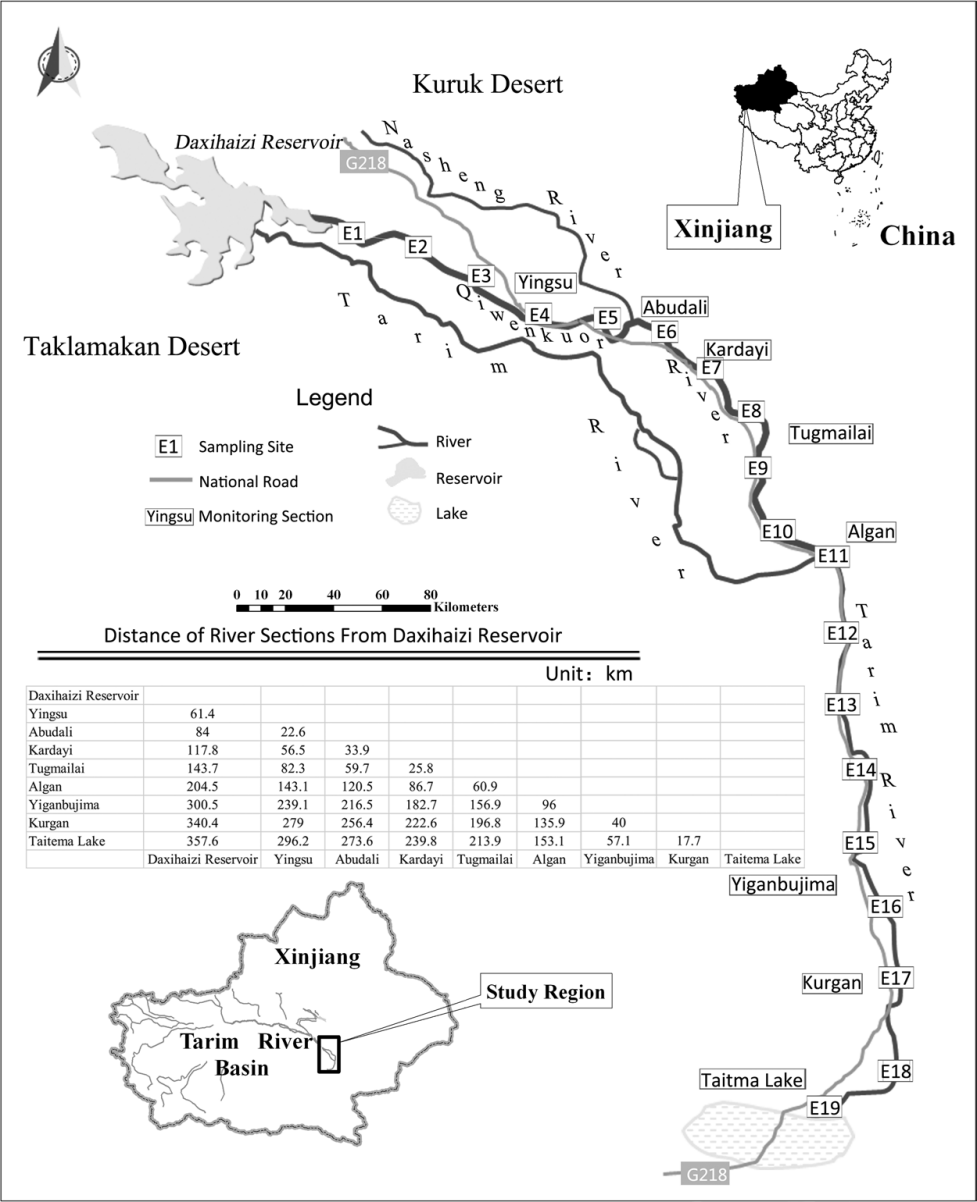
Sketch map and sampling sites distribution in study area.

**Figure 2:**
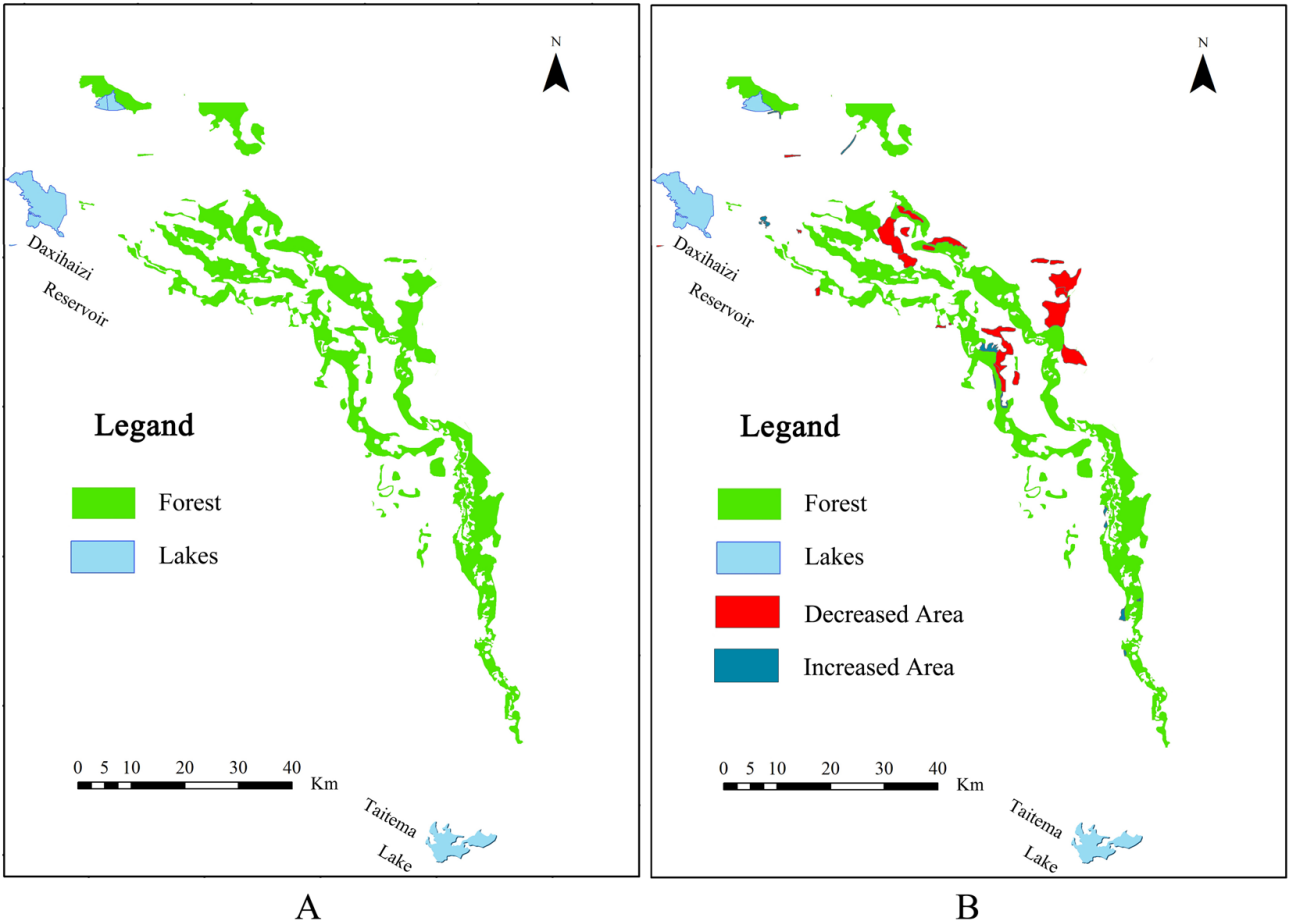
The spatial distribution of *P*. *euphratica* forests in the lower reaches of the Tarim River (**A**) represents observations from 2000, (**B**) represents observations from 2015, red and delft blue patches are decreased and increased areas of *P*. *euphratica* forest, respectively, from 2000 to 2015).

In addition, from our field investigations in lower reaches of Tarim River (as shown in Fig. 1), we observed two incomprehensible phenomena. The first one is that not a single *P*. *euphratica* tree (even dead trees) exists along the riverbank within 70 km stretch from the Kurgan section to Taitema Lake (Fig. 2), where water resources are relatively abundant. This phenomenon also occurs along the nearly 10 km section from the Daxihaizi Reservoir to Akdun. The other phenomenon is that as the EWDP is implemented, river flooding very frequently happens near the river banks^19^. Thousands of *P*. *euphratica* seedlings emerge from the surface soil in flooded areas after the floodwater recedes, while some non-flooded areas do not contain one single *P*. *euphratica* seedling, even the groundwater table is at a favourable level.

The aims of this research are to analyse the main factors influencing *P*. *euphratica* regeneration and determine how soil salinity affects the distribution and regeneration of *P*. *euphratica* forest. This will be accomplished by investigating the distributions of *P*. *euphratica* in response to the salinity of surface soils variations, exploring the effect of surface soil salinity on *P*. *euphratica* by seed germination experiments, and analysing the influence of river flooding on the salinity of surface soils through river flooding experiments. Our objectives are to reveal the mechanism of *P*. *euphratica* forest regeneration in the lower reaches of the Tarim River and explore the efficient ecological water delivery modes and effects of flooding on ecological restoration to guide the operation of the EWCP efficiently.

## Physical Settings

The Tarim River Basin (34.20°–43.39°N, 71.39°–93.45°E) is located in southern part of Xinjiang, China, and drains an area of 102 × 104 km^2^, out of which 66% is part of a desert range. The Tarim River eventually terminates in Taitema Lake, which surrounds the Tarim River basin from the west, north and east. Our study site is near Lop Nor, comprising a 428 km section of the lower reaches of the Tarim River, from Daxihaizi Reservoir to Taitema Lake. Throughout the watershed, the climate follows the cold-arid desert pattern of warming. Mean annual air temperature at this site is 18.5 °C, and the maximum and minimum temperature appear in July and December, respectively. Mean annual precipitation varies from 17.4 to 42.0 mm, but the annual potential evaporation is up to 3000 mm. Warm shrub and semi-shrub desert comprise the zonal vegetation. The focal species, *Populus euphratica* and *Tamarix*, occur throughout the study site, and *Phragmites australis*, *Alhagi sparsifolia*, and *Karelinia caspica* are among the herbaceous plants^42^. Within the study region, *P*. *euphratica* is a perennial species. Mature *P*. *euphratica* have great tolerance to drought and salinity. *P*. *euphratica* protects the development of oases, stabilizes river channels, maintains the ecological balance of river basins, and fertilizes the forest soil. This species has widely scattered populations that occur throughout the entire Tarim River Basin. It is extremely common along riparian areas in the lower reaches of the Tarim River, depending upon water availability. The main vegetation distribution is different between flooded and non-flooded sites.

## Methods

### Data resources

In September of 2015, nineteen 100 m-wide transects (E1 to E19 in) were established at average intervals of 20 km along the river channel in flooded and non-flooded areas, overlaying the lower reaches of the Tarim River. The length of each transect extended 100 m until the disappearance of *P*. *euphratica*. One hundred and five sampling sites of 100 m × 100 m were chosen at different distances from the river channel. (Fig. 1)

#### Field studies

Field studies were based on 105 grids of cells at different distances from the river channel. Three random squared 10 m × 10 m subplots were placed within the first 100 m × 100 m plots along the river channel. All the random subplots in the first 100 m range were 20, 50 and 100 m away from the river channel, for a total of 45 points. For each plot and subplot, we listed all desert plant species, taking note whether each species had at least one individual. For each plot, we recorded vegetation height (defined as the measure in cm of the individual plant), total vegetation cover (visually estimated in percentage values), diameter at breast height (DBH), and crown diameter. In each subplot, we surveyed the number of *P*. *euphratica* seedlings.

#### Soil sampling

Soil sampling was also performed in September of 2015. The surface soils (0–5 cm) were collected according to the national soil inventory method^43,44^. Soils were collected in different directions using a hand soil auger from 3 randomly selected 5 cm × 5 cm spots within each plot and subplot. The total number of soil quadrats was 135, including 19 quadrats without vegetation. Soil samples were air dried, crushed, and passed through a 2 mm mesh sieve. The salt content of all soil samples was analysed using a suspension with a 1:5 soil:water ratio. The conductivity of the soil extract was determined by a DDS-307A conductivity metre.

### Experimental design

#### Seed germination

To address the influence of salinity on seed germination, the initial tests of seed germinability were conducted in September 2014. The *P*. *euphratica* seeds were collected at the beginning of September 2014. Nine saline water treatments at different concentrations (i.e., 0, 0.05, 0.1, 0.2, 0.3, 0.4, 0.5, 0.6 and 0.7 mol ‧ L^−1^.) were applied. Each experimental unit consisted of 100 seeds. Each treatment was replicated 3 times. Seeds were placed on moist blotter paper on Petri dishes in incubators. This study was carried out for 10 days in a growth chamber with 12 hr of light and 12 hr of darkness with a stationary temperature of 30 °C. The experimental control temperature was identical to the topsoil temperature during July and September in the lower reaches of the Tarim River. Each Petri dish was checked twice a day to observe the germination conditions; the date and numbers of germinated seeds were recorded each time.

#### Flooding disturbance

The purpose of this experiment was to explore the influence that river flooding has on the salinity of surface soils. According to the ecological water conveyance data acquired, we divided the flooding factors (frequency, duration) into four grades in the downstream reaches of the Tarim River (Table 1), combined with the actual river flooding conditions recorded by field investigations in the study area.

**Table 1:** Gradient divisions at monitoring sections in the Lower Tarim River.

Mode	Few	Multiple	Scour	Waterlogging
Flooding frequency (times ‧ a^−1^)	1–2	3–5	6–10	once or more
Flooding duration (d ‧ a^−1^)	10–20	21–50	5–10	70–90

This experiment was performed in late August 2014. For every flooded sample site, we collected three soil samples in the flooded area and three soil samples in the non-flooded area nearby (we considered the non-flooded area as the original situation), 5 cm × 5 cm and 5 cm deep, to measure the surface soil salinity. The total number of soil samples was 114. The treatments of the soil samples in the laboratory were the same as those described in 3.1. Then, we measured eight ions of the soil extract: carbonate and bicarbonate were determined by potentiometric titration; the chlorine ion was determined by nitrate radical titration method; the sulfate ion was determined by EDTA-indirect titrimetric methods; calcium and magnesium ions were determined by EDTA complexometric titration; and sodium and potassium ions were determined by flame photometric detector.

In every soil sampling site, we randomly placed 3 vegetation quadrats, with size 1 m × 1 m, 2 m × 2 m, or 5 m × 5 m, to investigate the plant species and growth conditions, and recorded the species, heights, diameters at breast height (DBH), numbers and coverage-at-large.

### Data processing

In this study, the diffusion and aggregation indices were calculated by field investigation data. Indices such as the diffusion index (*DI*), mean crowd (m^*^), “patchiness” index (*PAI*), negative binomial index (*K*), Cassie index (*CA*), and Morisita index (*I*_*δ*_) were used to calculate the distribution patterns and aggregation intensities of *P*. *euphratica* populations. The calculation of the diffusion index (*DI*) is as follows:

1 $$DI=\frac{{S}^{2}}{\overline{x}}$$

2 $${S}^{2}=\frac{1}{n-1}\times \sum _{i=1}^{n}({x}_{i}-\overline{x})$$

where *S*^2^ is the variance of population abundance, and it’s calculation method is shown in formula (2); $\overline{x}$ is the mean value of population abundance; n is the numbers of quadrats; *x*_*i*_ is the number of *P*. *euphratica* in *i*^th^ quadrat. If *DI* = 1, the population is distributed randomly; if *DI* > 1, the population is distributed aggregately; and if *DI* < 1, the population is distributed uniformly^45,46^. To test the significance of the deviation of *DI* from the Poisson Distribution, a t-test is utilized in this paper using the formula:

3 $$t=(DI-1)/\sqrt{\frac{2}{\sqrt{n-1}}}$$

If the checking standards are salient ($|t|\ge |{t}_{0.05}|$ at the 95% level; $|t|\ge |{t}_{0.01}|$ at the 99% level), the distribution result is credible; otherwise it is not.

The calculations of the mean crowd (m^*^) and “patchiness” indices (*PAI*) are as follows:

4 $${m}^{\ast }=\overline{x}+(\frac{{S}^{2}}{\overline{x}}-1)$$

5 $$PAI={m}^{\ast }/\overline{x}$$

where m^*^ represents the mean intensity of the crowd. If *PAI* = 1, the population is distributed randomly; if *PAI* > 1, the population is distributed aggregately; and if *PAI* < 1, the population is distributed uniformly^47,48^.

The calculations of the negative binomial index (*K*) and the Cassie index (*CA*) are as follows:

6 $$K=\frac{{\overline{x}}^{2}}{{S}^{2}-\overline{x}}$$

where *K* is used to represent the aggregation intensity of a population, and the smaller *K* is, the higher the intensity of the aggregation.

7 $$CA=\frac{1}{K}$$

If *CA* = 0, the population is distributed randomly; if *CA* > 0, the population is distributed aggregately; and if *CA* < 0, the population is distributed uniformly^46,49^.

The calculation of the Morisita index (*I*_*δ*_) is as follows:

8 $${I}_{\delta }=n\frac{\sum _{i=1}^{n}{x}_{i}({x}_{i}-1)}{N(N-1)}$$

where *N* is the total number of *P*. *euphratica*, and other indices are the same as those in formula (2). If *I*_*δ*_ = 1, the population is distributed randomly; if *I*_*δ*_ > 1, the population is distributed aggregately; and if *I*_*δ*_ < 1, the population is distributed uniformly^50^. The significance of *I*_*δ*_ could be calculated by F-test, as below:

9 $$F=[{I}_{\delta }(\sum _{i=1}^{n}{x}_{i}-1)+N-\sum _{i=1}^{n}{x}_{i}]/(N-1)$$

The reference *F*_0_ = *F* (*n*_1_, *n*_2_), where n_1_ = n-1 and n_2_ = ∞. If *F* < *F*_0_, the population is distributed aggregately; and if *F* > *F*_0_, the population is distributed randomly.

### Statistical analysis

Descriptive statistical analyses for the mean, standard deviation, median, minimum and maximum values, the coefficient of variation (CV), kurtosis and skewness were applied to the results from soil samples from 2014 and 2015. We used the software package SPSS 20.0 for Windows for all the conventional statistical analyses (SPSS Inc., Armonk, NY, USA).

## Results

### Spatial distribution of *P. euphratica* in the lower reaches of the Tarim River

The river channel of the lower reaches of the Tarim River (from Daxihaizi Reservoir to Taitema Lake) was revitalized after implementation of the Ecological Water Conveyance Project (EWCP). On the southern and northern sides of the river bank along the lower reaches of the Tarim River, the population of *P*. *euphratica* was unevenly distributed (Fig. 2). The distribution of the *P*. *euphratica* pattern was observed within a range of 2 kilometres away from the river channel. Until the year of 2015, some patches of *P*. *euphratica* which are far away from the river channel were in decline compared to their state in 2000, and some portions of *P*. *euphratica* forest have appeared where river flooding occurred. Generally, the decreased area is much larger that the increased area (Fig. 2-B).

**Figure 3:**
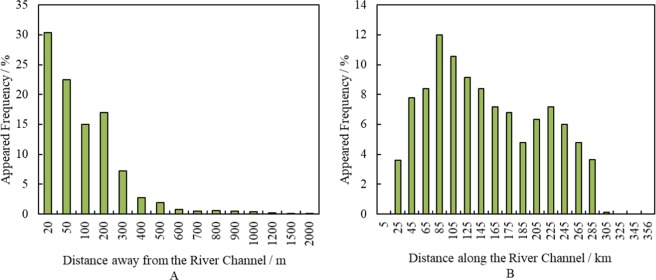
The frequency of occurrence of *P*. *euphratica* at different distances away from/along the river channel in the lower reaches of the Tarim River.

A total of 5050 individuals were found from the 105 sections of the 19 sites. The numbers of individuals were numerically dominated by mature trees (54.9%), followed by young trees (40.0%) and dead trees (5.1%). Figure 3 illustrates the frequency of occurrence of *P*. *euphratica* following the different distances along the lower reaches of the Tarim River. The frequency of occurrence of *P*. *euphratica* decreased significantly (*P* < 0.001) with increasing distance from the river channel. The sites with forest vegetation or non-forest vegetation can easily be seen from Fig. 3. There was no single *P*. *euphratica* within the distances from 0–25 km and 305–358 (accurately 357.6) km of the Daxihaizi reservoir. The lowest frequency of occurrence was found at 305 km.

The variation in the distribution of different age-groups of *P*. *euphratica* could reflect the changes in environmental conditions and interruptions by anthropogenic activities. In most natural plant populations, vegetative reproduction is mainly dispersed around the maternal plant; young populations show an aggregated distribution. Aggregation decreased along with tree ageing. However, the opposite spatial distribution patterns of *P*. *euphratica* growth stages were found in the lower reaches of the Tarim River (Table 2).

**Table 2:** Distribution patterns of *P*. *euphratica* populations in the lower reaches of the Tarim River.

Tree age	*DI*	*PAI*	*I*_*δ*_	*C*_*A*_	*m* ^*^	*K*	*t*	*F*	Distribution patterns
Seedlings	1.32	2.13	2.19	1.13	0.61	0.88	2.22	1.33	Aggregated
Young trees	1.14	1.29	1.31	0.29	0.60	3.46	0.93	1.14	Aggregated
Middle-aged trees	1.37	1.46	1.42	0.36	1.14	2.17	2.47	1.32	Aggregated
Mature trees	1.75	1.72	1.75	0.72	1.79	1.39	5.18	1.77	Aggregated

Affected by river flooding, *P*. *euphratica* seedlings were mainly distributed in clusters within 50 m from the river channel. Although all indices of young trees indicated they were distributed aggregately, this was not significant (t = 0.93). Middle-aged and mature trees were showed highly aggregated patterns at the range of 2 km away from the river channel. The reason why *P*. *euphratica* showed an aggregated distribution in the lower reaches of the Tarim River was the long-term diversion of the river arising from groundwater recession, and where the groundwater depth was relatively shallow, *P*. *euphratica* survived.

### Soil salinity effects on the spatial distribution of *P. euphratica*

A total of 405 soil samples were collected from both naturally flooded and non-flooded sites. The surface soil in the lower reaches of the Tarim River exhibited high salinity (Table 3). The sampling point elevations ranged from 793 m to 805 m, with estimated soil electrical conductivity of saturation-extract from 1.12 ms/cm up to 39.76 ms/cm. The mean value of the electrical conductivity of soil solutions was 6.51 ms/cm, belonging to a moderately-highly saline soil. The CV for soil salinity was 104.36%, which represents an excessive level of variation.

**Table 3:** Statistical features of surface soil salinity.

Sample size	Minimum	Maximum	Mean	Median	Variance	CV%	Skewness	Kurtosis
405	1.12 ms/cm	39.76 ms/cm	6.51 ms/cm	4.58 ms/cm	46.16	104.36	2.08	5.47

**Figure 4:**
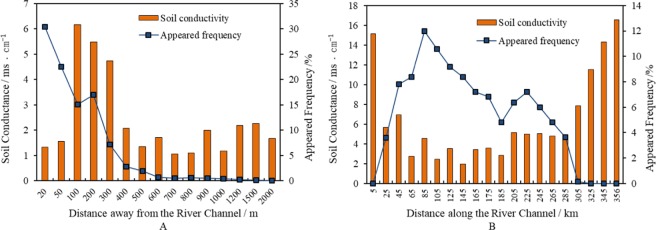
The relationships between soil salinity and frequency of occurrence of *P*. *euphratica* at different distances away from/along the river channel in the lower reaches of the Tarim River.

The relationships between soil salinity and occurrence patterns of *P*. *euphratica* are presented in Fig. 4. In the range of 50 metres away from the river channel, the surface soil salinity (i.e., soil conductivity) was lower than 2 ms/cm (Fig. 4-A). There was a sharp increase in soil salinity when the distance exceeded the range of 100 metres, then soil salinity slightly declined until the distance of 300 metres away from the river channel. The soil conductivity declined sharply as the distance exceeded 300 metres away from the river channel, with a minimum of 1.06 ms/cm and a maximum of 2.26 ms/cm. Clear relationships were not found between the occurrence patterns of *P*. *euphratica* and soil salinity, while the occurrence patterns showed a significant decline with increasing distance from the river channel.

The spatial evolution of surface soil conductivity along the river channel was studied by collecting 57 soil samples at the distance of 100 m away from the river channel. Surface soil conductivity showed higher values at the range of 0–5 km and 305–358 km along the river channel as shown in Fig. 4, because of the intense soil water evaporation under high groundwater-level conditions at the close range and the high salt contents in lacustrine sediments at the farther range. By contrast, surface soil conductivity showed moderate variations in the range of 25–285 km. The variation of *P*. *euphratica* occurrence along the river channel showed that where the soil conductance was higher than 8 ms/cm, the frequency of *P*. *euphratica* dropped to 0.

### Soil salinity and seed germination rates of *P. euphratica*

Plant growth is highly affected by abiotic stress factors. Among various abiotic stresses, salinity and drought are the most destructive. *P*. *euphratica* is the only tree species naturally distributed at the edge of barren deserts or semi-barren deserts worldwide and it is well known for its high tolerance to salinity and atmospheric drought. Mature *P*. *euphratica* have been shown to secrete salts from the base of their tree trunks.

**Figure 5:**
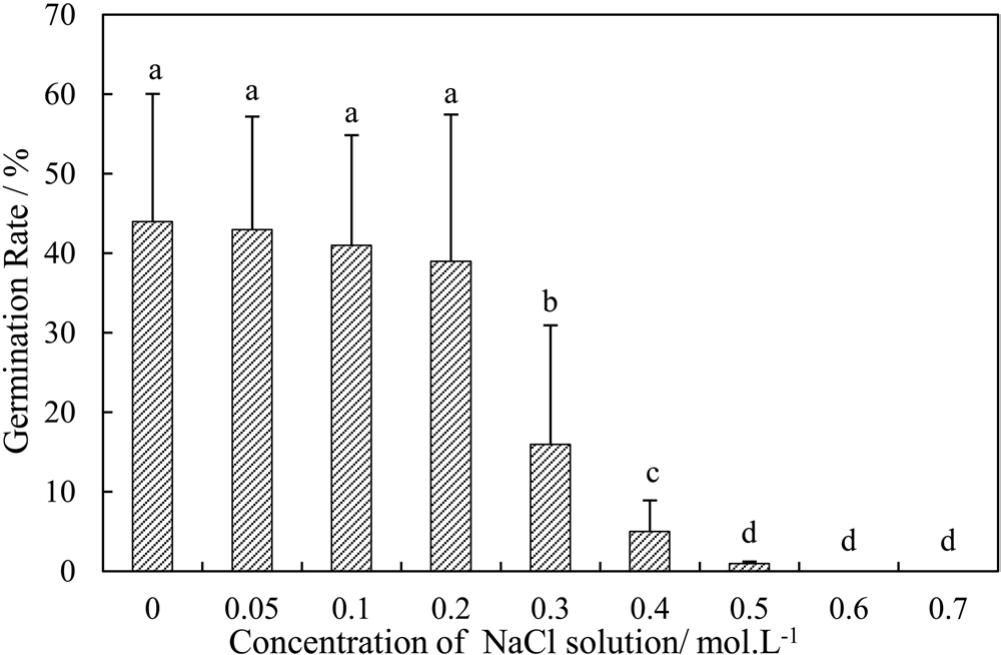
The germination rate of *P*. *euphratica* seeds under different salt concentrations.

Soil salinity is a major abiotic stress influencing plant productivity. The spatiotemporal variability of soil salinity and its effects on *P*. *euphratica* seeds were investigated. Figure 5 shows the effects of different soil salinity levels on *P*. *euphratica* seed germination. Seed germination of *P*. *euphratica* decreased with increasing salinity level. Germination of *P*. *euphratica* seeds was not significantly affected by 0.2 mol l^−1^ NaCl, however, under 0.3 mol l^−1^, germination decreased by 64% in comparison to the control (Fig. 5). At 0.6 mol l^−1^ NaCl, the seeds were unable to germinate.

### Flooding effects on surface soil salinity

**Figure 6:**
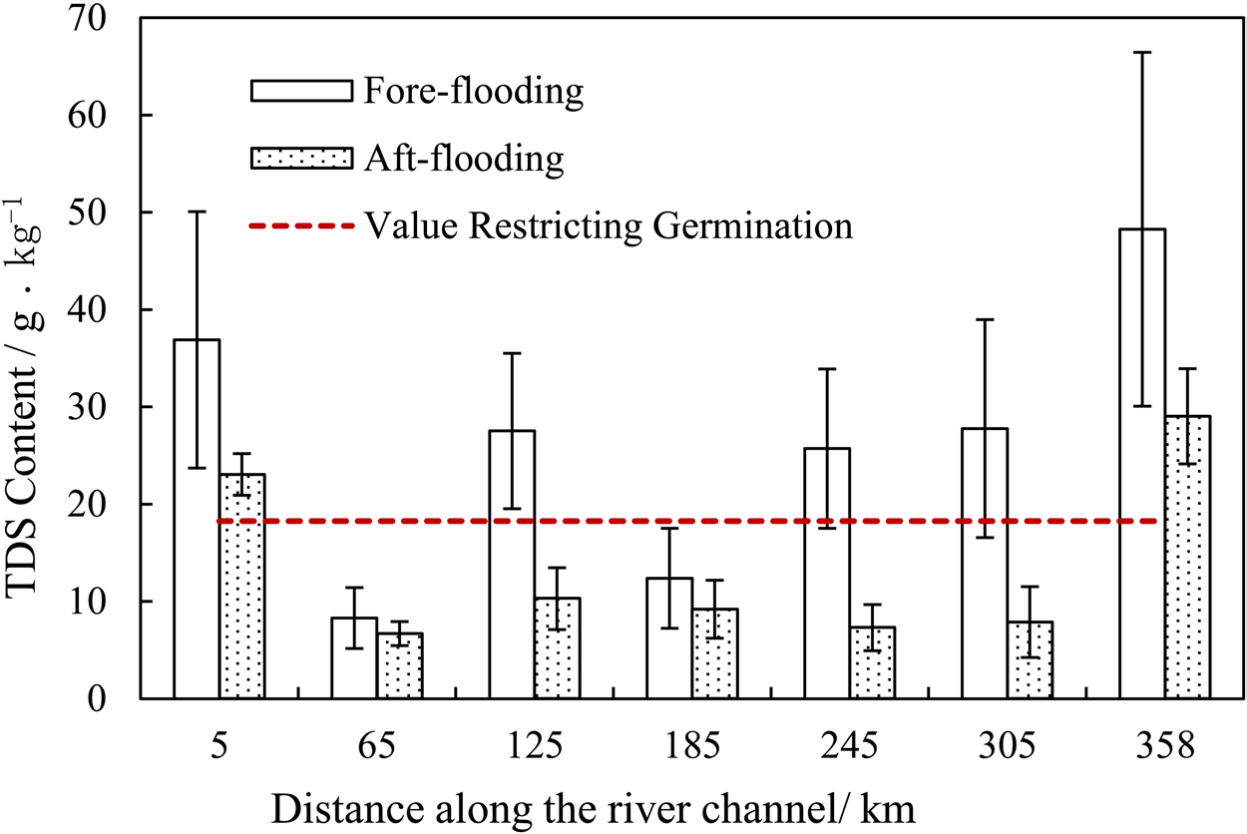
The changes of soil salinity fore-and-aft flooding.

The one-way ANONA (Table 4) showed a significant variance in soil salinity among the different studied sites (p < 0.001). The total soil salt contents at the seven sections were significantly affected by flooding and differed by distance along the river channel. Total soil salinity reduced by 38%, 20%, 63%, 26%, 72%, 71% and 40% at the seven sections after flooding, respectively (Fig. 6).

**Table 4:** One-way ANOVA of flooding effects on soil salinity.

Soil salt	Sum of squares	df	Mean Square	F	Sig.
Between Groups	89.627	1	89.627	8.294	0.006
Within Groups	583.539	112	10.806		
Total	673.166	113

**Figure 7:**
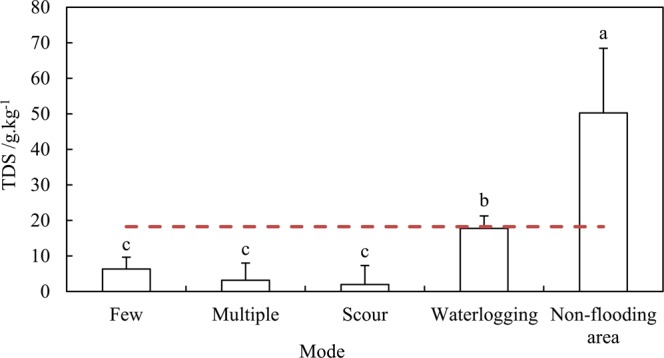
The influence of flooding mode on TDS. Note: The different letters indicate significant differences at the 0.05 level; Horizontal orange line represents the value restricting germination.

The salinity level was significantly affected by the hydrologic regime. A significant difference in TDS content variations occurred with different flooding modes (p < 0.05)(Fig. 7). At the non-flooded area, the TDS content significantly decreased among four hydrological regimes. When the flooding frequency increased, the TDS declined insignificantly, while the TDS was significantly higher than other flooding grades when the duration of flooding was over 70 days. The salinity of the surface soil was lowest for the scour regime.

This indicates that a few occurrences of river flooding could greatly reduce the salinity of surface soils, and many flooding events, even scouring, do not have clear effects for decreasing salinity. The salinity was much higher for the waterlogging regime.

## Discussion

### Surface soil salinity effects on *P. euphratica* distribution

Groundwater depth determines the distribution and growth of *P*. *euphratica*^51,52^. It should also be noted that salinization of soil is also severe in this area, which is neglected under severe pressure of water shortage. Mature *P*. *euphratica* trees is known to be relaticely tolerance to water and salt stress. Wang^53^ indicates that when the salt content of soil reaches 22.5 g/kg, *P*. *euphratica* trees still grow well, which they suggest is related to the capacity for “salt-secreting”. While the seeds, seedlings and young trees do not have this kind of ability^53^. According to our research, soil salinity influences the distribution of *P*. *euphratica* by restricting the germination of seeds. This would explain the observation that where the soil conductivity is relatively high, the occurrence rate of *P*. *euphratica* is extremely low, as shown in Fig. 4B. However, as shown in Fig. 4A, the frequency of occurrence of *P*. *euphratica* declines with the decrease of soil salinity, it can be explained by the groundwater and restricts the growth of *P*. *euphratica*.

**Figure 8:**
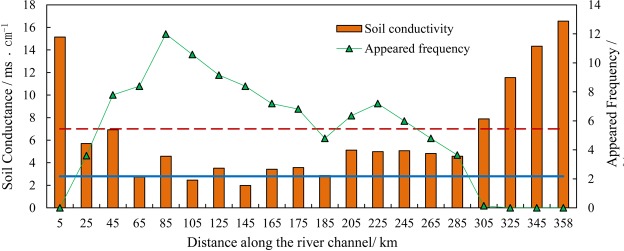
The spatial distribution of the surface soil conductance in the lower reaches of the Tarim River. Note: red dash line represents the value of 7 ms/cm, blue straight line represents the value of 2.8 ms/cm.

According to the germination experiment, when the concentration of the solution was higher than 0.2 mol/L, the germination rate declined significantly, when the concentration exceeded 0.5 mol/L, the germination of *P*. *euphratica* was severely curtailed. The concentrations of 0.2 mol/L and 0.5 mol/L are equivalent to the soil conductivity of 2.8 ms/cm and 7 ms/cm, respectively. Following the Fig. 4-B, we illustrated this with two horizontal lines as shown in Fig. 8.

Figure 8 illustrates that where soil conductivity is higher than 7 ms/cm, the frequency of *P*. *euphratica* diminished drastically and drops to 0%. The high salinity of the surface soil has restricted the germination of *P*. *euphratica* seeds. This is why we did not observe large areas of *P*. *euphratica* in two places (0–25 km and 305–358 km along the river channel in the lower reaches of the Tarim River). We could not conclude there is not a clear and significant correlation with the soil conductivity and frequency of *P*. *euphratica* when the soil conductivity is below 2.8 ms/cm and between 2.8 and 7 ms/cm (P = 0.31). Thus, it is not necessary to reduce the soil conductivity below 2.8 ms/cm to guarantee a high germination rate. The restrictive effect of soil salinity will be relieved when the soil conductivity is reduced below 7 ms/cm.

### Flooding effects on *P. euphratica* forest restoration

Connell^54^ proposed the famous intermediate disturbance hypothesis (IDH), which indicates that an intermediate disturbance could maintain high biodiversity^54^. The IDH was originally developed and proposed that an intermediate disturbance could help restore natural vegetation^55–58^. As one of the disturbance modes, river flooding is widely used in vegetation reconstruction. Zhang^59^ conducted a river flooding experiment to restore *Tamarix* in Celle County and received rewards from the United Nations^59^. In Germany, Vervuren conducted river flooding experiments in the Rhine River during flood events^60^. For hyper-arid areas, the lower reaches of the Tarim River, a typical inland river which has experienced long-term ecological degradation arising from the diversion of the river, river flooding might have more significant value. The regeneration of *P*. *euphratica* forest has a close relationship with river flooding^61^. River diversion and flooding are the main reasons why the world’s largest *P*. *euphratica* forest developed in the Tarim River basin.

According to our research, the range of river flooding is approximately 50 metres from the riverbank. In the range of 50 metres, river flooding happens frequently, where the soil conductivity is relatively low. Within the range of 100 to 300 metres, the groundwater depth is relatively shallow because of runoff recharging, which lead to phreatic evaporation and salinity accumulation in surface soils. When the distance exceeds 300 metres, there is less salinity accumulation arising from by phreatic evaporation, because the groundwater depth is relatively deep, and the soil conductivity has no significant difference from that in the range of 50 metres. This indicates that even though the soil salinity is much more prominent in the study area, it does not exceed the restrictive value for the germination of *P*. *euphratica* seeds. Still the main reason why large areas of *P*. *euphratica* could not survive is unfavourable water condition.

The effects of river flooding on *P*. *euphratica* forest reconstruction are as follows. (1) During the period of seed germination, river flooding could reduce the surface soil salinity and increase the soil moisture, that guarantee seed germination and seedling survival. (2) During the period of seedling growth to young tree, river flooding can recharge soil water to guarantee its normal growth. When the roots of young trees are able to reach groundwater, maintaining proper groundwater depth is enough to protect the growth. This paper proposes the theory that maintaining the suitable groundwater depth is sufficient for preserving the *P*. *euphratica* forest, river flooding is necessary if we want to restore the *P*. *euphratica* forest.

According to the results of the river flooding experiment, soil salinity could be reduced significantly after river flooding, even merely once or twice per year. However, if the volume of flood water is large, leading to waterlogging, the water table will be raised and salt accumulation will occur in surface soils by water and soil evaporation.

### Transfer mode of ecological water conveyance

As of October 2018, the EWCP has been conducted 19 times during the last 18 years since 2000, 7.44 billion m^3^ of ecological water has been transferred to the lower reaches of the Tarim River. The data is shown in Table 5.

**Table 5:** Previous eco-water conveyance in the Daxihaizi section in the lower reaches of the Tarim River.

Order	Mode	Stage	Period	Volume (10^8^ m^3^)	Arrival Location
1	single channel	Emergent	May to Jul., 2000	0.99	Kardayi
2	single channel	Emergent	Nov., 2000 to Feb., 2001	2.27	Algan
3	double channels	Emergent	April to Nov., 2001	3.82	Taitema Lake
4	double channels & branches	Emergent	Jun. to Nov., 2002	3.31	Taitema Lake
5	double channels & patches	Emergent	Mar. to Nov., 2003	6.25	Taitema Lake
6	single channel	Emergent	Apr. to Jun., 2004	1.02	Taitema Lake
7	double channels	Normal	Apr. to Nov., 2005	2.84	Taitema Lake
8	single channel	Normal	Sept. to Nov., 2006	1.96	Kurgan
9	single channel	Normal	Sept. and Oct., 2007	0.14	Kardayi
10	single channel	Normal	Nov. and Dec., 2009	0.11	Kardayi
11	double channels	Normal	Jun. to Nov., 2010	3.64	Taitema Lake
12	double channels & branches	Normal	Jan. to Nov., 2011	8.52	Taitema Lake
13	double channels & branches	Normal	Apr. and Man, 2012	6.67	Taitema Lake
14	double channels & branches	Normal	Apr. to Nov., 2013	4.88	Taitema Lake
15	double channels & branches	Normal	Jun. to July, 2014	0.07	Taitema Lake
16	double channels & branches	Normal	Aug. to Nov.,2015	4.22	Taitema Lake
17	double channels & branches	Normal	Aug. to Oct., 2016	6.76	Taitema Lake
18	double channels & branches	Normal	May to No., 2017	12.15	Taitema Lake
19	double channels & branches	Normal	Feb. to Oct. 2018	4.80	Taitema Lake
Total				74.42	

Note: this table was re-organized according to Deng *et al*.^62^.

Ecological water conveyance in the lower reaches can be divided into two stages (Table 5). The first stage (2000–2004) was emergent water delivery, diverting water from the Kaidu-Kongque River and Bosten Lake downstream to the Tarim River. This historical diversion of the river flow have been terminated the zero-flow event from 1970s. The second stage (2005-present), normal water delivery, water from three sources (i.e., Aksu River, Yarkand Darya, and Hotan Rivers) flowing to the lower reaches of the Tarim River have been gradually replaced the Bosten Lake emergency water supply. Because of the flooding period and comprehensive management of the Tarim River basin, the ecological water conveyance become routine.

From the period of ecological water conveyance in Table 4, it is worth noting that the EWDP does not have a well-planned schedule, instead of diverting water according to the inflows of source rivers and upper streams, leading to variations in flooding period and volume among years. During high-flow years, large volumes of ecological water were transferred to Taitema Lake, resulting in a 100 km^2^ of wet area, which leads to a large amount of useless evaporation and severe salt accumulation. Ecological water conveyance without proper planning, only aimed at fulfilling the task of water diversion. This makes no sense regarding to a better ecological restoration of the lower reaches of the Tarim River. Deng *et al*.^62^ proposed a new water transfer patterns of branches and patches based on a single channel and double channels water delivery, with the aim of water flowing through the river channel and recharging groundwater^62^. The purpose of developing these two new patterns is to create the condition of water flooding to guarantee the growth and regeneration of the desert riparian *P*. *euphratica* forest.

According to previous studies, the ripe germination period of *P*. *euphratica* seeds is June to August, and the seeds can only be maintained for 20 days^63^. In order for the EWCP to be effective, combined with the effects of river flooding on soil salinity, we propose the EWCP should implement water flooding twice every year during June to August, with a 10- to 15-day duration each time. This agrees with the flooding mode we proposed previously based on the community ecology^24^. In addition, in order to accomplish large-scale ecological restoration using limited water, a comprehensive and suitable irrigation rotation schedule is needed^64^.

## Conclusions

This paper manipulated the interactions between the hydrologic regime and salinity level through investigations into the relationships between *P*. *euphratica* distributions and surface soil salinity and experiments of seed germination and river flooding. As revealed by the research carried out in the lower reaches of the Tarim River, we answered the scientific questions regarding,(1) how soil salinity affects the distribution of *P*. *euphratica*, and (2) further investigated the role that river flooding plays in the process of *P*. *euphratica* restoration. In the lower reaches area, *P*. *euphratica* trees mainly spread along the river channel within 2 km, with increasing vertical distance to the river channel, the number of trees declines. The salinity of the surface soil has a close relationship with the germination of *P*. *euphratica* seeds. The germination of *P*. *euphratica* seeds was severely curtailed, when the soil conductivity exceeds 7 ms/cm. We found that river flooding is a pre-condition for seed germination which can ameliorate water conditions and reduce salinity, as revealed by the seed germination and river flooding experiments. We experimentally evaluated suitable river flooding modes for the restoration of the desert riparian forest, which may be incorporated into later ecological water conveyance process and river flooding mode, i.e., twice every year, during June to August, with a 10- to 15-day duration each time. Protection and restoration of the desert riparian forest can be slow and difficult, such an efficient ecological prescription should both meet the needs of supplying ecological water for growth and restoration of the desert riparian forest and high-efficient utilization of water for national food production.
